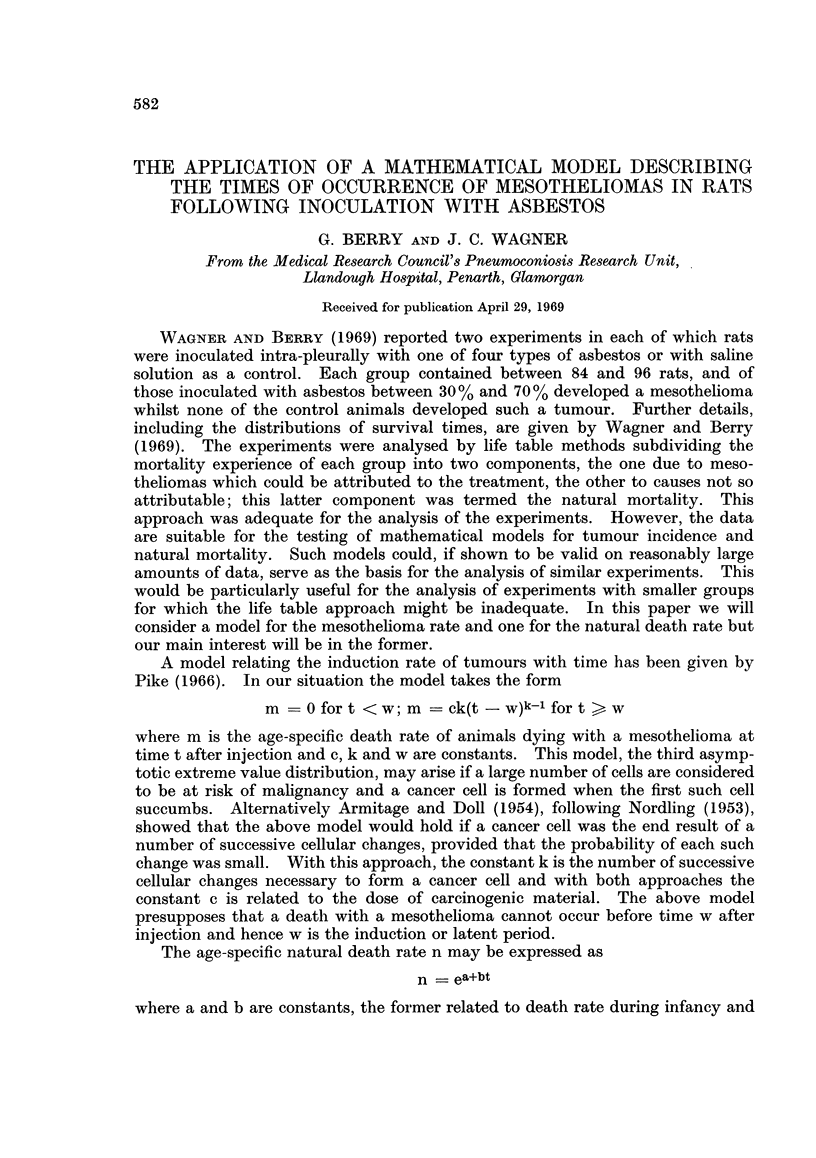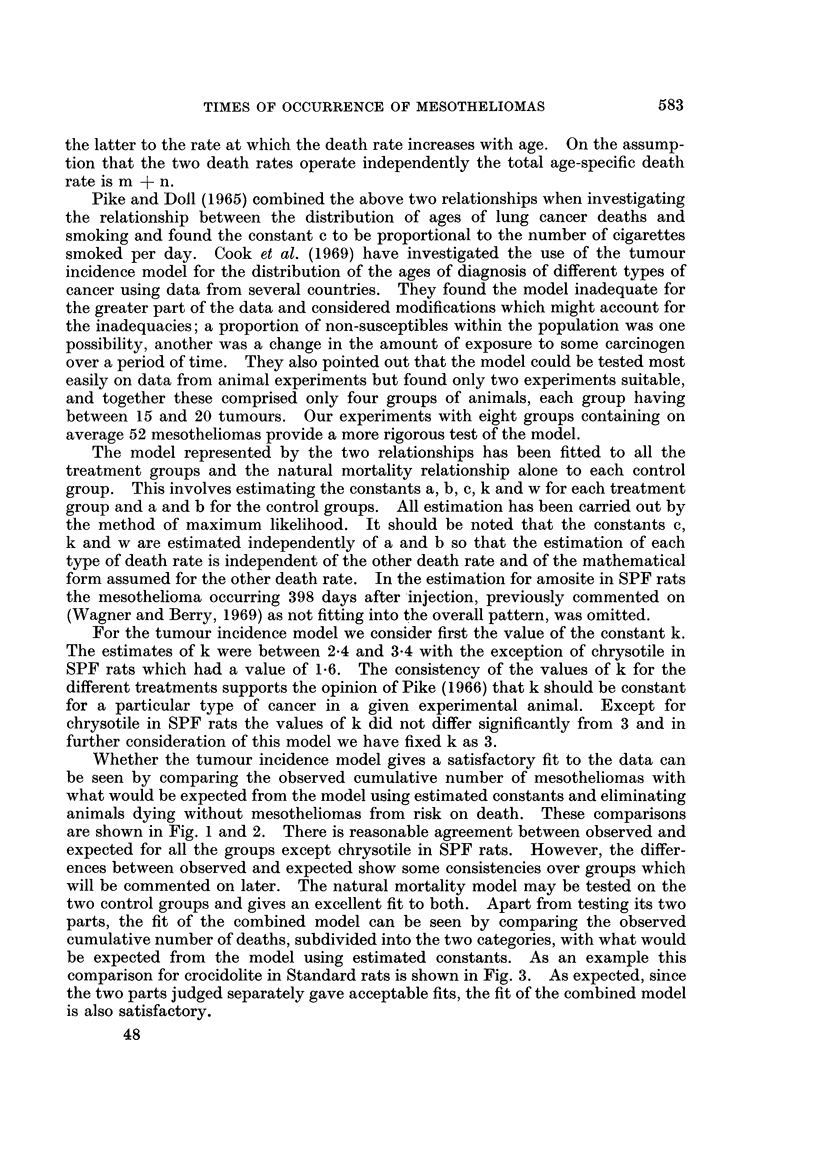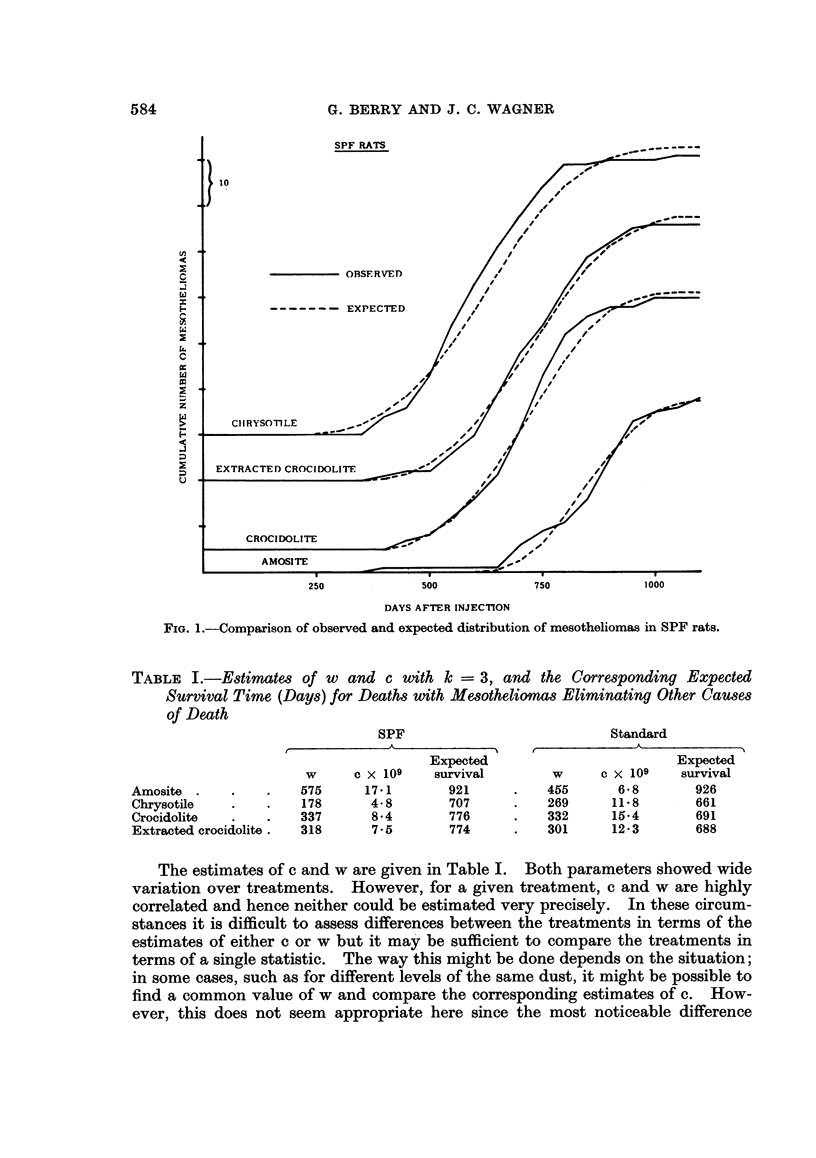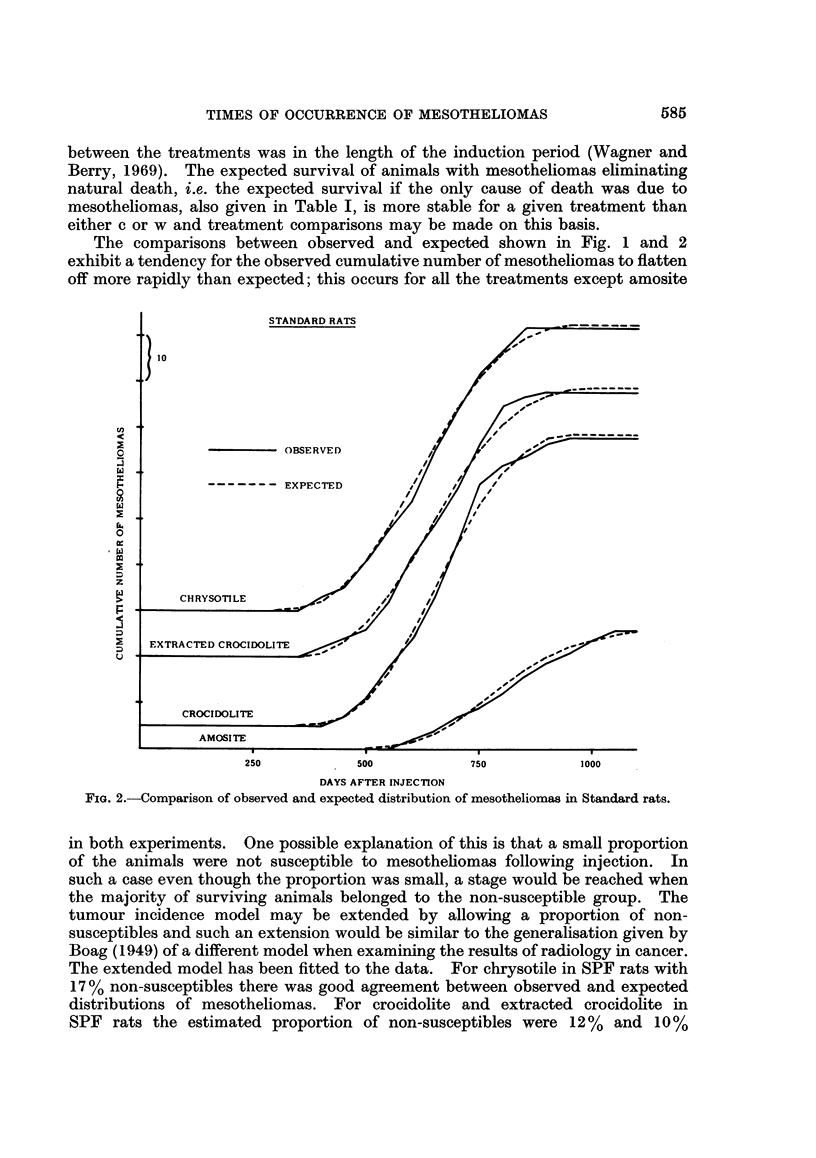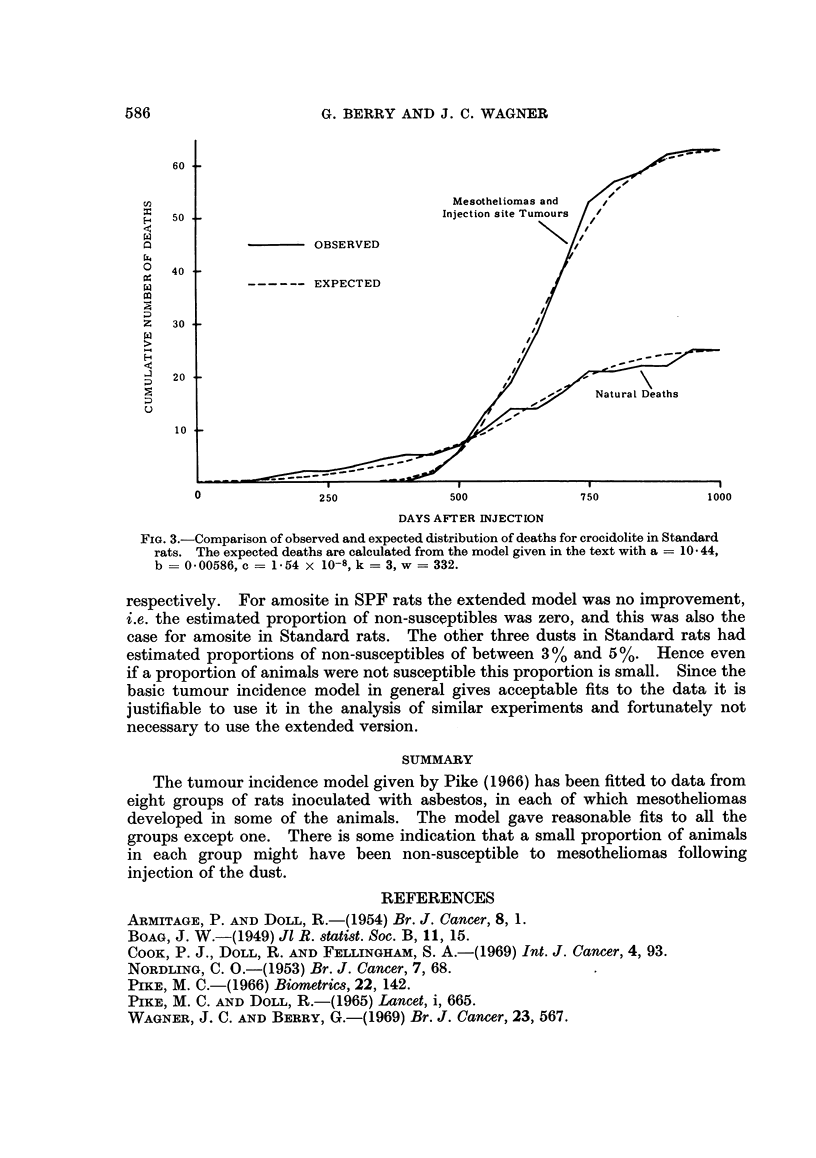# The application of a mathematical model describing the times of occurrence of mesotheliomas in rats following inoculation with asbestos.

**DOI:** 10.1038/bjc.1969.71

**Published:** 1969-09

**Authors:** G. Berry, J. C. Wagner


### THE APPLICATION OF A MATHEMATICAL MODEL DESCRIBING THE TIMES OF OCCURRENCE OF MESOTHELIOMAS IN RATS FOLLOWING INOCULATION WITH ASBESTOS

G. BERRY AND J. C. WAGNER

*From the Medical Research Council's Pneumoconiosis Research Unit, Llandough Hospital, Penarth, Glamorgan*

Received for publication April 29, 1969

WAGNER AND BERRY (1969) reported two experiments in each of which rats were inoculated intra-pleurally with one of four types of asbestos or with saline solution as a control. Each group contained between 84 and 96 rats, and of those inoculated with asbestos between 30% and 70% developed a mesothelioma whilst none of the control animals developed such a tumour. Further details, including the distributions of survival times, are given by Wagner and Berry (1969). The experiments were analysed by life table methods subdividing the mortality experience of each group into two components, the one due to mesotheliomas which could be attributed to the treatment, the other to causes not so attributable; this latter component was termed the natural mortality. This approach was adequate for the analysis of the experiments. However, the data are suitable for the testing of mathematical models for tumour incidence and natural mortality. Such models could, if shown to be valid on reasonably large amounts of data, serve as the basis for the analysis of similar experiments. This would be particularly useful for the analysis of experiments with smaller groups for which the life table approach might be inadequate. In this paper we will consider a model for the mesothelioma rate and one for the natural death rate but our main interest will be in the former.

A model relating the induction rate of tumours with time has been given by Pike (1966). In our situation the model takes the form

$$m = 0 \text{ for } t < w; \; m = ck(t - w)^{k-1} \text{ for } t \geqslant w$$

where m is the age-specific death rate of animals dying with a mesothelioma at time t after injection and c, k and w are constants. This model, the third asymptotic extreme value distribution, may arise if a large number of cells are considered to be at risk of malignancy and a cancer cell is formed when the first such cell succumbs. Alternatively Armitage and Doll (1954), following Nordling (1953), showed that the above model would hold if a cancer cell was the end result of a number of successive cellular changes, provided that the probability of each such change was small. With this approach, the constant k is the number of successive cellular changes necessary to form a cancer cell and with both approaches the constant c is related to the dose of carcinogenic material. The above model presupposes that a death with a mesothelioma cannot occur before time w after injection and hence w is the induction or latent period.

The age-specific natural death rate n may be expressed as

$$n = e^{a+bt}$$

where a and b are constants, the former related to death rate during infancy and the latter to the rate at which the death rate increases with age. On the assumption that the two death rates operate independently the total age-specific death rate is m + n.

Pike and Doll (1965) combined the above two relationships when investigating the relationship between the distribution of ages of lung cancer deaths and smoking and found the constant c to be proportional to the number of cigarettes smoked per day. Cook *et al.* (1969) have investigated the use of the tumour incidence model for the distribution of the ages of diagnosis of different types of cancer using data from several countries. They found the model inadequate for the greater part of the data and considered modifications which might account for the inadequacies; a proportion of non-susceptibles within the population was one possibility, another was a change in the amount of exposure to some carcinogen over a period of time. They also pointed out that the model could be tested most easily on data from animal experiments but found only two experiments suitable, and together these comprised only four groups of animals, each group having between 15 and 20 tumours. Our experiments with eight groups containing on average 52 mesotheliomas provide a more rigorous test of the model.

The model represented by the two relationships has been fitted to all the treatment groups and the natural mortality relationship alone to each control group. This involves estimating the constants a, b, c, k and w for each treatment group and a and b for the control groups. All estimation has been carried out by the method of maximum likelihood. It should be noted that the constants c, k and w are estimated independently of a and b so that the estimation of each type of death rate is independent of the other death rate and of the mathematical form assumed for the other death rate. In the estimation for amosite in SPF rats the mesothelioma occurring 398 days after injection, previously commented on (Wagner and Berry, 1969) as not fitting into the overall pattern, was omitted.

For the tumour incidence model we consider first the value of the constant k. The estimates of k were between 2·4 and 3·4 with the exception of chrysotile in SPF rats which had a value of 1·6. The consistency of the values of k for the different treatments supports the opinion of Pike (1966) that k should be constant for a particular type of cancer in a given experimental animal. Except for chrysotile in SPF rats the values of k did not differ significantly from 3 and in further consideration of this model we have fixed k as 3.

Whether the tumour incidence model gives a satisfactory fit to the data can be seen by comparing the observed cumulative number of mesotheliomas with what would be expected from the model using estimated constants and eliminating animals dying without mesotheliomas from risk on death. These comparisons are shown in Fig. 1 and 2. There is reasonable agreement between observed and expected for all the groups except chrysotile in SPF rats. However, the differences between observed and expected show some consistencies over groups which will be commented on later. The natural mortality model may be tested on the two control groups and gives an excellent fit to both. Apart from testing its two parts, the fit of the combined model can be seen by comparing the observed cumulative number of deaths, subdivided into the two categories, with what would be expected from the model using estimated constants. As an example this comparison for crocidolite in Standard rats is shown in Fig. 3. As expected, since the two parts judged separately gave acceptable fits, the fit of the combined model is also satisfactory.

FIG. 1.—Comparison of observed and expected distribution of mesotheliomas in SPF rats.

TABLE I.—*Estimates of w and c with k = 3, and the Corresponding Expected Survival Time (Days) for Deaths with Mesotheliomas Eliminating Other Causes of Death*

| | SPF | | | Standard | | |
|---|---|---|---|---|---|---|
| | w | c × $10^9$ | Expected survival | w | c × $10^9$ | Expected survival |
| Amosite | 575 | 17·1 | 921 | 455 | 6·8 | 926 |
| Chrysotile | 178 | 4·8 | 707 | 269 | 11·8 | 661 |
| Crocidolite | 337 | 8·4 | 776 | 332 | 15·4 | 691 |
| Extracted crocidolite | 318 | 7·5 | 774 | 301 | 12·3 | 688 |

The estimates of c and w are given in Table I. Both parameters showed wide variation over treatments. However, for a given treatment, c and w are highly correlated and hence neither could be estimated very precisely. In these circumstances it is difficult to assess differences between the treatments in terms of the estimates of either c or w but it may be sufficient to compare the treatments in terms of a single statistic. The way this might be done depends on the situation; in some cases, such as for different levels of the same dust, it might be possible to find a common value of w and compare the corresponding estimates of c. However, this does not seem appropriate here since the most noticeable difference between the treatments was in the length of the induction period (Wagner and Berry, 1969). The expected survival of animals with mesotheliomas eliminating natural death, *i.e.* the expected survival if the only cause of death was due to mesotheliomas, also given in Table I, is more stable for a given treatment than either c or w and treatment comparisons may be made on this basis.

The comparisons between observed and expected shown in Fig. 1 and 2 exhibit a tendency for the observed cumulative number of mesotheliomas to flatten off more rapidly than expected; this occurs for all the treatments except amosite

FIG. 2.—Comparison of observed and expected distribution of mesotheliomas in Standard rats.

in both experiments. One possible explanation of this is that a small proportion of the animals were not susceptible to mesotheliomas following injection. In such a case even though the proportion was small, a stage would be reached when the majority of surviving animals belonged to the non-susceptible group. The tumour incidence model may be extended by allowing a proportion of non-susceptibles and such an extension would be similar to the generalisation given by Boag (1949) of a different model when examining the results of radiology in cancer. The extended model has been fitted to the data. For chrysotile in SPF rats with 17% non-susceptibles there was good agreement between observed and expected distributions of mesotheliomas. For crocidolite and extracted crocidolite in SPF rats the estimated proportion of non-susceptibles were 12% and 10%

FIG. 3.—Comparison of observed and expected distribution of deaths for crocidolite in Standard rats. The expected deaths are calculated from the model given in the text with a = 10·44, b = 0·00586, c = $1·54 \times 10^{-8}$, k = 3, w = 332.

respectively. For amosite in SPF rats the extended model was no improvement, *i.e.* the estimated proportion of non-susceptibles was zero, and this was also the case for amosite in Standard rats. The other three dusts in Standard rats had estimated proportions of non-susceptibles of between 3% and 5%. Hence even if a proportion of animals were not susceptible this proportion is small. Since the basic tumour incidence model in general gives acceptable fits to the data it is justifiable to use it in the analysis of similar experiments and fortunately not necessary to use the extended version.

### SUMMARY

The tumour incidence model given by Pike (1966) has been fitted to data from eight groups of rats inoculated with asbestos, in each of which mesotheliomas developed in some of the animals. The model gave reasonable fits to all the groups except one. There is some indication that a small proportion of animals in each group might have been non-susceptible to mesotheliomas following injection of the dust.

### REFERENCES

ARMITAGE, P. AND DOLL, R.—(1954) *Br. J. Cancer*, **8**, 1.
BOAG, J. W.—(1949) *Jl R. statist. Soc.* B, **11**, 15.
COOK, P. J., DOLL, R. AND FELLINGHAM, S. A.—(1969) *Int. J. Cancer*, **4**, 93.
NORDLING, C. O.—(1953) *Br. J. Cancer*, **7**, 68.
PIKE, M. C.—(1966) *Biometrics*, **22**, 142.
PIKE, M. C. AND DOLL, R.—(1965) *Lancet*, i, 665.
WAGNER, J. C. AND BERRY, G.—(1969) *Br. J. Cancer*, **23**, 567.